# Genetic Adaptations in Mudskipper and Tetrapod Give Insights into Their Convergent Water-to-Land Transition

**DOI:** 10.3390/ani11020584

**Published:** 2021-02-23

**Authors:** Juwan Kim, Chul Lee, DongAhn Yoo, Heebal Kim

**Affiliations:** 1 Interdisciplinary Program in Bioinformatics, Seoul National University, 1 Gwanak-ro, Gwanak-gu, Seoul 08826, Korea; madapada@snu.ac.kr (J.K.); swear0712@gmail.com (C.L.); dongahn.yoo@gmail.com (D.Y.); 2 Department of Agricultural Biotechnology and Research Institute of Agriculture and Life Sciences, Seoul National University, 1 Gwanak-ro, Gwanak-gu, Seoul 08826, Korea; 3 eGnome, Inc., 26 Beobwon-ro 9-gil, Songpa-gu, Seoul 05836, Korea

**Keywords:** vertebrate land invasion, tetrapod, comparative genomics, *Periophthalmus*, evolution, positive selection, amino acid substitution

## Abstract

**Simple Summary:**

Mudskippers are amphibious fish that spend most of their lives on mudflats. The morphological and physiological adaptations of mudskippers resemble water-to-land transition of ancient tetrapod, yet comparative studies on the genetic backgrounds underlying the adaptation are still limited. The aim of this study is to compare genome-wide signatures of positive selection and gene family evolution between mudskipper and tetrapods. Here, we performed comparative genomics analysis between terrestrial tetrapods, coelacanth, mudskipper, and aquatic fish species. As a result, we discovered genes with selective signature in tetrapod and mudskipper lineages associated with immune responses, mitochondrial oxidative phosphorylation, kidney development, and DNA repair processes. In both tetrapod and mudskipper linages, we found convergent contraction of the gene family encoding βγ-crystallins that are found in the lens of vertebrate eye and involved in the refraction of the light. Our results present similar genetic adaptations in mudskipper and tetrapod lineages, which independently adapted to land environment.

**Abstract:**

Water-to-land transition has been independently evolved in multiple vertebrate lineages including the most recent common ancestor of tetrapod and multiple fish clades, and among them, mudskippers uniquely adapted to the mudflat. Even though physiological and morphological adaptation of mudskippers is thought to resemble that of the ancestral tetrapod, it is unclear if they share genome-wide evolutionary signatures. To detect potential signatures of positive selection in mudskipper and tetrapods, we analyzed 4118 singleton orthologues of terrestrial tetrapods, coelacanth, mudskipper, and fully aquatic fishes. Among positively selected genes identified in mudskipper and tetrapod lineages, genes involved in immune responses, mitochondrial oxidative phosphorylation, and kidney development were detected. On the other hand, tetrapod-specific and mudskipper-specific positively selected genes were functionally enriched for DNA repair processes, which could be associated with higher exposure to UV light. We also performed gene family analysis and discovered convergent contraction of eight gene families, including βγ-crystallin coding genes in both tetrapod and mudskipper lineages. Findings of this study suggest the similar genetic adaptation against environmental constraints between the ancient tetrapod and mudskippers for their land adaptation.

## 1. Introduction

Water-to-land transition refers to the macro-evolution of aquatic organisms adapting to the terrestrial environment, supported by multiple pieces of evidence, including the emergence of our well-known ancestor-like species, *Tiktaalik*, in the Late Devonian Epoch [1]. Even though land adaptation of ancient tetrapods, so called vertebrate land invasion, is one of the most famous and well-studied examples of water-to-land transition, it is thought that transition from aquatic to terrestrial environments has evolved in multiple lineages independently including turtles [2] and other invertebrates such as snails and arthopods [3]. Especially, multiple fish species have developed adaptation to the terrestrial environment independently [4] that varies from temporal terrestriality of water of climbing perch to the seasonal terrestriality of mangrove rivulus to the fully amphibious lifestyle of mudskipper, showing that adaptation to the land environment is not just a single historical event but has been repeatedly happening in multiple lineages.

Mudskippers including *Periophthalmus magnuspinnatus* are amphibious fishes that spend most of their time on mudflats. Their unique traits well demonstrate physiological and morphological adaptations against ecological constraints of the terrestrial environment. They acquired eyes on the top of their head, which enable them to feed and recognize predators on land. They periodically retract their eyes beneath their suborbital dermal cups, which works like blinking of tetrapods [5,6]. Towards the stronger effect of gravity on land, mudskippers evolved their terrestrial locomotive behaviors (so called ‘crutching’) with highly modified pectoral fins that resemble the locomotion of a seal [7,8]. Different oxygen levels between land and water made them a bimodal respirator that using cutaneous respiration for 50 to 60% of aerial respiration [9]. Recent studies revealed convergent thirst mechanisms between mudskippers and mammals, and mudskippers’ ability to use acoustic communication out of water [6,10]. They are also highly resistant to ammonia unlike typical aquatic fish, which is regarded as an adaptation to excretion out of water [11]. Intertidal mudflat is one of the most drastically changing environment in terms of salinity, temperature, and oxygen level, leading to several adaptations in mudskippers. Mitochondrion-rich cell of mudskippers maintains their body-fluid osmolality by regulating ion transportation [12]. As an adaptation to temperature changes, they rely on solar heat, and hibernate in burrows during the cold seasons. Occasionally, the burrows become hypoxic, and then mudskippers gulp fresh air and replace it with the hypoxic air [13]. Additionally, hemoglobins of mudskippers show high oxygen binding affinities, similar to those of other hypoxia-tolerate fishes, which may be associated with the mudskippers’ adaptation to the hypoxic condition as well [14].

The unique adaptations of mudskippers have led to various studies on mudskippers to elucidate the mechanisms of vertebrate land invasion [7,15,16]. Several studies supported the intertidal origin of ancient tetrapod’s land invasion [17,18] which resembles the habitats of mudskippers, but it is still controversial whether the invasion was held in the tropical lowlands that accompanied seasonal drying or in intertidal mudflats [15,19]. However, one thing we know is that both mudskippers and ancestor of tetrapod should have faced similar ecological challenges in their land adaptations. Regarding the terrestrial adaptation of tetrapods, there have been some studies to propose genetic signatures associated with essential physiological modifications of ancestral tetrapods such as limb development (as known as fin-to-limb transition), changes in chemoreceptors, and evolution of nitrogen excretion mechanism based on comparative genomics approaches [20,21]. Similarly, as reviewed by You et al. [22], several studies reported unique genetic characteristics of mudskippers related to their land adaptation with the genomics and transcriptomics approaches [23,24,25]. Nevertheless, there still have been few studies to compare the genetic signature of land adaptation between mudskipper and ancestor of tetrapods, which can provide candidate genes or insights into biological processes related to the land adaptation of the two distinct vertebrate lineages.

Adaptation to a new environment is often accompanied by the functional changes of protein sequences by amino acid substitutions [26]. Thus, signatures of potential positive selection can be detected via comparative genomics approach based on the ratio of nonsynonymous to synonymous substitution rates (dN/dS ratio, or ω) in the singleton ortholog genes [27]. Along with the singleton orthologues, adaptation can be also driven by the evolution of gene families [28,29], which can be detected by estimating expansion and contraction of gene families among different species [30,31].

Here, we performed comparative genomics analysis with terrestrial tetrapods, coelacanth, mudskipper, and fully aquatic teleost species. We focused on amino acid substitutions with the signature of positive selection and rapid evolution of gene families in land-adapting lineages on various terrestrial traits.

## 2. Materials and Methods

### 2.1. Collection, Alignment, and Trimming of Singleton Orthologues

The coding sequences of 12 species, including 4 terrestrial tetrapod species (human: *Homo sapiens*, mouse: *Mus musculus*, cattle: *Bos taurus*, and chicken: *Gallus gallus*), coelacanth (*Latimeria chalumnae*), giant-fin mudskipper (*Periophthalmus magnuspinnatus*), and 6 fully aquatic teleostean fishes (zebrafish: *Danio rerio*, orange clownfish: *Amphiprion percula*, Eastern happy: *Astatotilapia calliptera*, Japanese medaka: *Oryzias latipes*, Indian glassy fish: *Parambassis ranga* and Amazon moly: *Poecilia formosa*) were downloaded from BioMart in Ensembl database release 98 [32] to investigate the signature of positive selection on the singleton (one to one) orthologues (Table 1). Three standards were considered for selecting species set. First, model animals (human, mouse, zebrafish: the model system of developmental biology, and Amazon molly: the model system of carcinogenesis) were selected to facilitate functional inference of genes. Second, species of our interest were included such as coelacanth representing aquatic lobe-finned fishes and mudskipper. Lastly, the remaining species were selected based on the assembly quality (contig N50 > 1 Mbp). With custom Python code (version 3.6.9, htttp://www.python.org, accessed on 10 January 2021), we collected coding sequences (CDS) of 4185 singleton orthologues based on Ensembl ortholog. The CDS with lengths not in multiple of 3 were discarded and codon-wise alignment was performed with prank v.140603 [33] with the following options: -F and -codon. To eliminate the poorly aligned positions, we performed Gblocks v0.91b [34] with the following option -d = c for codon-wise trimming. After excluding the genes that failed to align or no sequences remaining after trimming, 4118 singleton orthologues were retained for the downstream analysis.

### 2.2. Identification of Positive Selection Related to Water-to-Land Transition

Tree of analyzed species was retrieved from TimeTree database [35] and unrooted by ETE 3 [36]. Three species nodes (*Poecilia formosa*, *Astatotilapia calliptera, and Periophthalmus magnuspinnatus*) not available in the database were replaced with the node of their sister taxa (*Poecilia reticulata*, *Haplochromis burtoni*, and *Periophthalmus argentilineatus*, respectively). To detect the signature of positive selection, we performed branch-site test with CodeML in Phylogenetic Analysis by Maximum Likelihood (PAML) package v4.9 [37] on 4118 singleton orthologues. The branch-site model A allows heterogeneous ω (the ratio of nonsynonymous to synonymous substitution rates) across sites and branches in its model (model = 2, NSsites = 2, and CodonFreq = 2), enabling the detection of positive selection along specified branches by comparing with a null model (model = 2, NSsites = 2, CodonFreq = 2, fix_omega = 1, and omega = 1) that assumes neutral or purifying selection across all sites and branches. Since there were two branches involving water-to-land transition (the branch with the common ancestor of tetrapod species and the mudskipper branch), we designed three different tests with model A, which assumes (1) positive selection on the tetrapods’ common ancestor branch, but not on the mudskipper branch (tetrapod model), (2) positive selection on the mudskipper branch, but not on the tetrapods’ common ancestor lineage (mudskipper model), and (3) positive selection on both branches (tetrapod-and-mudskipper model), respectively. Likelihood-ratio test (LRT) was performed to test for the alternative hypothesis, which assumes positive selection. We considered a gene is under potential positive selection if they showed ω > 1 for the site class 2 (ω_2_ > 1), with higher likelihood in model A than that of the null model (2∆L > 0), and adjusted *p*-values by false discovery rate (FDR) below 0.1 (FDR < 0.1). Based on the Ensembl gene id of human, functional analysis of the genes under positive selection was performed with ClueGO v2.5.7 [38] within the Cytoscape framework v3.6 [39] with using Benjamini-Hochberg corrected *p*-value threshold below 0.05.

### 2.3. Detection of Amino Acid Substitution under Positive Selection

To identify candidate amino acid substitutions related to the water-to-land transition, amino acid residues with a posterior probability > 0.95 by the Bayes empirical Bayes (BEB) [40] were first defined as positively selected sites (PSSs). Target-specific amino acid substitution (TAAS) analysis [41] compares amino acid residues and classifies the site into four classes if there are no overlapping amino acid residues between target and the remaining species set (Supplementary Figure S1). We performed TAAS analysis by changing target species to (1) mudskipper (corresponding to mudskipper model), (2) 4 tetrapod species (corresponding to tetrapod model), and (3) mudskipper plus the tetrapods (corresponding to tetrapod-and-mudskipper model), and next selected PSSs overlapping with the TAAS sites. To find exclusive amino acid substitution patterns between the target species and the remaining species of other teleostean fishes and tetrapods that were not included in our study, we investigated the amino acids alignment of orthologues retrieved from Ensembl release 98, checked if the exclusive amino acid substitution patterns found between target or remaining species accounted for more than 90% on the PSSs by custom Python code, and named them as exclusive amino acid substitutions under positive selection (EAASPS, Supplementary Figures S2–S4). Finally, domain search was performed by NCBI conserved domain search v.3.18 [42] with expected value < 0.01 and domain information from UniProt [43] to check whether PSSs are located within functional domain of each protein by using protein sequences of human as a query. Illustrator for Biological Sequences (IBS) v1.0 [44] was used for visualization.

### 2.4. Detection of Rapidly Evolving Gene Families

Out of the CDS of the 12 species and outgroup species (elephant shark: *Callorhinchus milii*) from Ensembl release 98, we selected the longest transcript of each gene as a representative transcript. For human, mouse, and zebrafish whose genome assembly include alternate scaffolds that represent haplotypes or strain-specific genomic regions, the genes on the alternate scaffolds were excluded by custom Python code. Based on Computational Analysis of gene Family Evolution (CAFE) workflow (https://hahnlab.github.io/CAFE/, accessed on 10 January 2021), we performed all-by-all protein BLAST [45] with the following option –seg = yes and -outfmt = 7. After finding clusters of similar sequences by mcl (http://micans.org/mcl/, accessed on 10 January 2021) [46,47], we downloaded the phylogenetic tree with divergence time from TimeTree database [35]. We performed CAFE [30,31] analysis (v.4.2.1, https://hahnlab.github.io/CAFE/, accessed on 10 January 2021) assuming equal evolutionary rate and *p*-value threshold for families below 0.05 to detect the gene families under rapid expansion or contraction involved in transition from water to land.

## 3. Results

### 3.1. Identifications and Classifications of Positively Selected Genes under Different Evolutionary Models Related to Water-to-Land Transition

**Figure 1:**
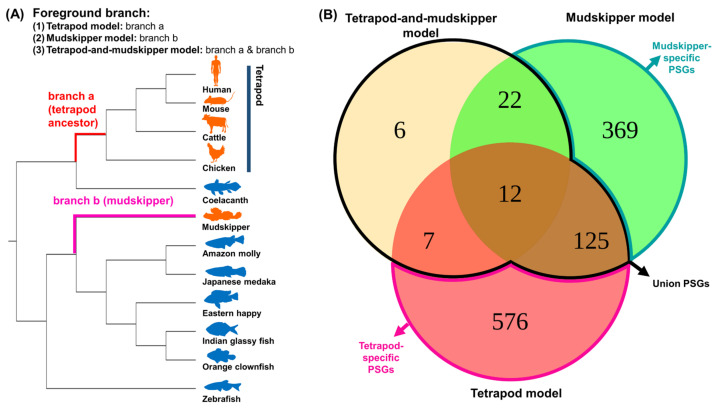
Different evolutionary models assuming positive selection on land-adapting lineages and the number of positively selected genes (PSGs). (**A**) Phylogeny was downloaded from TimeTree database [35]. Orange and blue indicate land-adapting and fully aquatic species, respectively. Red indicates the common ancestor branch of terrestrial tetrapod species and pink indicates mudskipper branch. For the branch-site test, three different alternative model A were tested by changing foreground branch (the branch under potential positive selection) to branch a (tetrapod model), branch b (mudskipper model), and both branches (tetrapod-and-mudskipper model). (**B**) The number of PSGs detected under threshold (FDR < 0.1, 2∆ L > 0, and ω_2_ > 1). Red, green, and yellow backgrounds indicate PSGs in tetrapod model, mudskipper model, and tetrapod-and-mudskipper model, respectively. Pink, green, and black lines indicate tetrapod-specific, mudskipper-specific, and union PSGs, respectively.

To detect the signatures of positive selection related to water-to-land transition in mudskipper and tetrapod ancestor lineage, we performed branch-site test with CodeML in PAML package [37] on 4118 singleton orthologous genes shared by four terrestrial tetrapod species, coelacanth, mudskipper, and six aquatic teleostean fishes. The test was performed three times by changing the foreground branch (branch under potential positive selection) to the most recent common ancestor of terrestrial tetrapods (tetrapod model, Supplementary Table S1), mudskipper (mudskipper model, Supplementary Table S2), and both lineages (tetrapod-and-mudskipper model, Supplementary Table S3) (Figure 1A).

The positively selected genes (PSGs) from the three models were classified into three different groups. We regarded PSGs that were uniquely detected in tetrapod or mudskipper model as the exclusive candidate genes for ancient tetrapod (tetrapod-specific PSGs) or mudskipper (mudskipper-specific PSGs), respectively. On the other hand, the signature of positive selection can be detected in the different residues of the same gene, depending on the foreground branch of each model. Thus, we regarded a gene as candidates for terrestrial adaptation on the both lineages (union PSGs) if the gene is detected in tetrapod-and-mudskipper model or detected in both tetrapod and mudskipper model. In this way, we detected 576 tetrapod-specific, 369 mudskipper-specific, and 172 union PSGs (FDR <0.1, 2△L > 0, ω2 >1, Figure 1B).

### 3.2. Amino Acid Substitutions on the Functional Domains in Union PSGs

**Figure 2:**
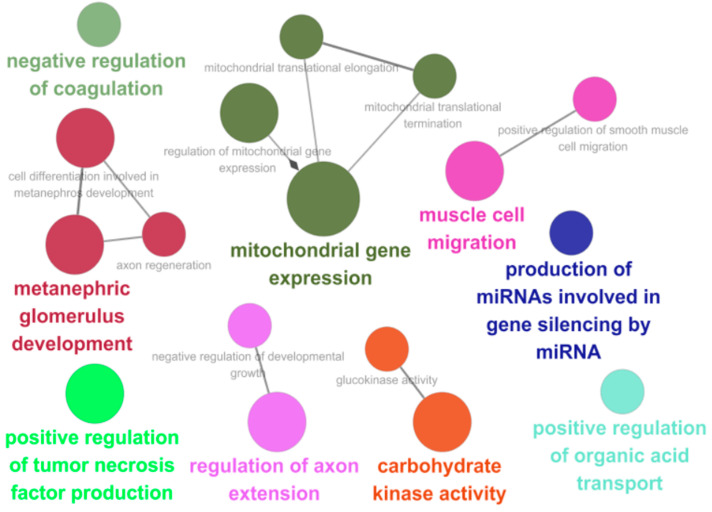
Functional annotation of union PSGs. The gene ontology network was constructed by ClueGO [38] with PSGs under FDR< 0.1, 2∆ L >0, and ω_2_ >1.

To list up the potential candidate genes for land adaptation of both lineages, we performed functional annotation of the union PSGs with ClueGO [38] and discovered significant functional enrichment in 17 biological processes within 9 groups of union PSGs that include “positive regulation of tumor necrosis factor production”, “mitochondrial gene expression”, and “metanephric glomerulus development” (Figure 2).

**Figure 3:**
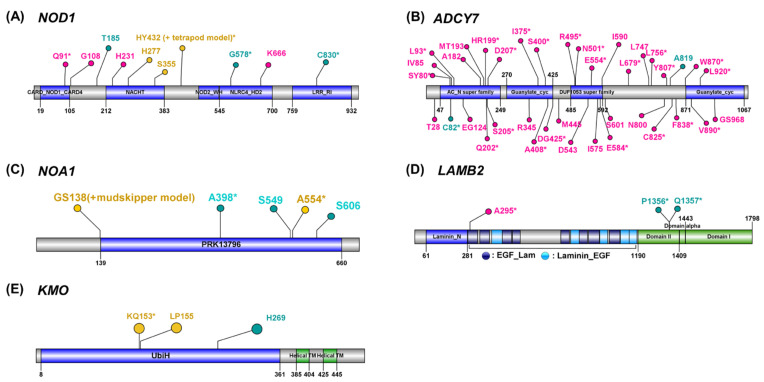
Positively selected amino acid substitutions in functional domains. Labels with the amino acid residue and the position of positively selected TAAS site of target species were annotated (yellow, red, green: a positively selected site detected from tetrapod-and-mudskipper model, tetrapod model, or mudskipper model, respectively). A label with asterisk indicates EAASPS (exclusive amino acid substitution under positive selection). Blue and green bars indicate domains or regions predicted by NCBI conserved domain search [42] or from UniProt [43], respectively.

If a specific amino acid substitution leads to the functional change, the exclusive pattern of amino acid substitution between the target (mudskipper, tetrapods, or both lineages depending on different evolutionary models) and the remaining species should be well conserved in the singleton orthologues of other vertebrates not included in the current analysis. Thus, based on protein alignment of orthologues, we investigated if the EAASPSs (see Section 2 and Supplementary Figures S2–S4) were also conserved in other aquatic fishes and tetrapods. Based on the functional enrichment test and literature reviews, we listed up the PSGs with EAASPSs as potential candidates involved in immune responses, mitochondrial oxidative phosphorylation, and kidney development, all of which are suggested as the biological processes potentially associated with land adaptation [3,22,23,48,49]. Nucleotide-binding oligomerization domain-containing protein 1 gene (*NOD1*) mediates innate and acquired immunity by activating the NF-κB signal pathway and promoting the expression of inflammatory cytokines to induce the resistance against the infection of bacteria [50,51]. We discovered 3 EAASPSs and 4 PSSs in *NOD1*′s functional domains and (Figure 3A), including H277 from tetrapod-and-mudskipper model that was not EAASPS but still showed highly conserved exclusive amino acid patterns (Supplementary Table S4) and within the NACHT domain that involves in MHC transcription activation [52]. Adenylate Cyclase 7 gene (*ADCY7*) which is involved in the regulation of immune responses [53] showed 1 and 22 EAASPSs in mudskipper and tetrapod models, respectively (Figure 3B). Its AC_N Superfamily domain that covers the N-terminal extracellular and transmembrane regions included six tetrapod model EAASPSs and single mudskipper model EAASPS. In addition, we discovered one mudskipper model EAASPS and one tetrapod-and-mudskipper model EAASPS within the same domain on Nitric Oxide Associated 1 gene (*NOA1*), which acts as an essential GTPase in mitochondrial protein synthesis [54] and is involved in the regulation of mitochondrial respiration [55] (Figure 3C). We detected two PSGs with EAASPSs involving the development of kidneys. Laminin subunit beta-2 gene (*LAMB2*) encodes for laminin protein, a major component of renal glomerular basement [56]. This gene included one tetrapod model EAASPS and two mudskipper model EAASPSs on separate domains, respectively (Figure 3D). We also detected one EAASPS from the tetrapod-and-mudskipper model on the functional domain of Kynurenine 3-mono-oxygenase (*KMO*) previously suggested as a causal factor of proteinuria [57] (Figure 3E).

### 3.3. Enrichment of DNA Repair Processes on Tetrapod-Specific and Mudskipper-Specific PSGs

Next, we investigated whether tetrapod-specific and mudskipper-specific PSGs were involved in similar biological processes, which can be considered as potential common biological processes related to their water-to-land transition. We performed functional annotation on the 369 mudskipper-specific and 576 tetrapod-specific PSGs (FDR < 0.1, 2△L > 0, ω_2_ > 1) and discovered that mudskipper-specific PSGs were significantly enriched with 135 biological processes clustering into 29 groups (Supplementary Table S5) including “negative regulation of double-strand DNA repair” and “positive regulation of viral life cycle”. In tetrapod-specific PSGs, we detected 105 biological processes in 29 groups (Supplementary Table S6) including “DNA repair”, like enrichment of DNA repair related processes in mudskipper-specific PSGs. Additionally, we detected immunity-related biological processes including “regulation of toll-like receptor 4 signaling pathway”, “leukocyte apoptotic process”, and “regulation of B cell proliferation” in tetrapod-specific PSGs as well.

**Figure 4:**
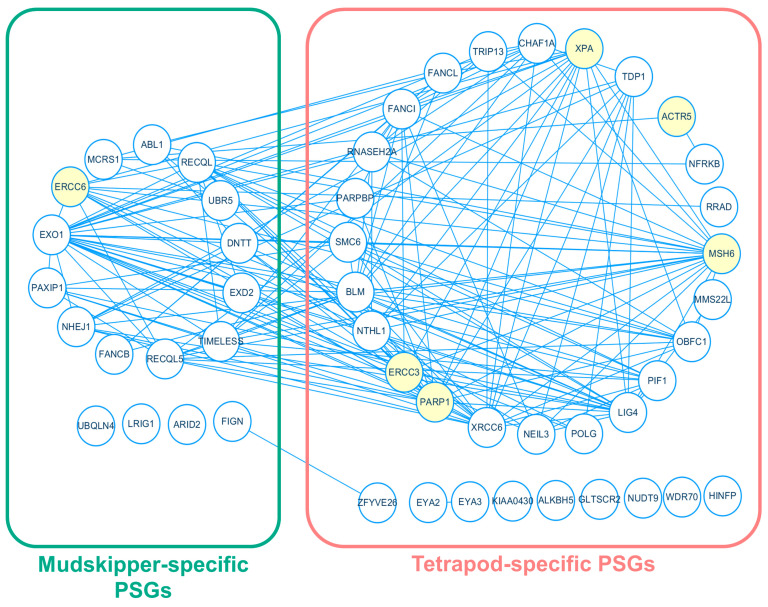
Protein-protein interaction network between tetrapod-specific and mudskipper-specific PSGs involved in “DNA repair”. Green and red indicates mudskipper-specific and tetrapod-specific PSGs, respectively. Yellow indicates the six PSGs involved in “response to UV”.

Based on the functional enrichment result, we focused on 30 mudskipper-specific PSGs in the gene ontology group represented as “negative regulation of double-strand DNA repair” and 101 tetrapod-specific PSGs in the gene ontology group represented as “DNA repair”, which was suggested as mudskippers’ potential adaptation to higher DNA damage from the prolonged exposure to UV light in a terrestrial environment [23]. To find associations among the PSGs, we performed a protein-protein interaction (PPI) network analysis based on STRING database v.11 [58] and discovered a single interaction network that includes 13 mudskipper-specific PSGs and 24 tetrapod-specific PSGs in “DNA repair” (Figure 4), which is a necessary process to repair the damaged DNA from exposures of UV light [59]. Inside the network, there were single mudskipper-specific PSG, *ERCC6*, and five tetrapod-specific PSGs-*PARP1*, *ERCC3*, *ACTR5*, *MSH6*, and *XPA* that were involved in “response to UV”. Except *ACTR5*, the five remaining PSGs formed a single PPI network and *ERCC6* had interactions with all other genes that were experimentally determined (Supplementary Figure S5). Thus, our result suggests potential adaptation of tetrapod and mudskipper in the genes related to DNA repair mechanism as a countermeasure of elongated exposure to direct UV light.

### 3.4. Convergent Contraction of βγ-Crystallin Gene Superfamily in Land-Adapting Species

**Figure 5:**
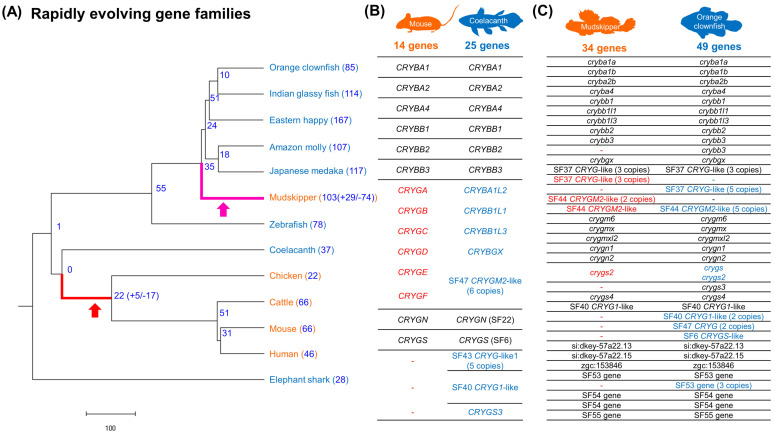
Rapidly evolving gene families in mudskipper and tetrapod ancestor lineages. (**A**) Phylogeny and the number of rapidly evolving gene families. Branch length indicates divergence time (million years ago). (**B**,**C**) Contraction of crystallin coding genes in mouse and mudskipper. Orthologous genes were within the same row. The genes without any symbol were annotated by (1) the description or (2) consensus name of their Ensembl protein family ID (PTHR11818_XXXX). Red and blue indicate the potential contraction of genes in terrestrial species and their orthologues in aquatic species, respectively.

Rapid contraction and expansion of gene families were investigated with café [30,31]. We detected 17 contracted and 5 expanded gene families in the tetrapod lineage and 74 contracted and 29 expanded gene families in mudskipper lineage, respectively (Figure 5A). Among them, eight types of gene families were both rapidly contracted in mudskipper and tetrapod lineages (Supplementary Table S7). Based on functional annotation of the eight types of gene families, we discovered rapid contraction in the βγ-crystallin gene superfamily that encode water-soluble structural proteins in the vertebrate lens. The gene family was further investigated by comparing the number of genes included in the 25 Ensembl protein families that were included in the βγ-crystallin gene superfamily defined by CAFE (Supplementary Table S8). Terrestrial tetrapods showed far less number of genes (9–14) compared to aquatic coelacanth (26) and similarly, mudskipper showed fewer genes, of 34, compared to majority of aquatic fishes (49.8 genes in average) except Japanese medaka. Next, we focused on the number of the genes coding γM-crystallin that are abundant in the lenses of fish [60]. Based on the zebrafish annotation, which contains the largest number of γM-crystallin genes, five Ensembl protein family (PTHR11818_SF37, PTHR11818_SF40, PTHR11818_SF41, PTHR11818_SF44, and PTHR11818_SF53) were regarded as γM-crystallin coding gene families. Similar with the result above, there were no γM-crystallin coding genes in terrestrial tetrapods while two were observed in coelacanth. Mudskipper showed fewer γM-crystallin coding genes (16 genes) than most aquatic fishes (28.5 genes in average) as well, except Japanese medaka with 16 genes (Supplementary Table S8).

**Figure 6:**
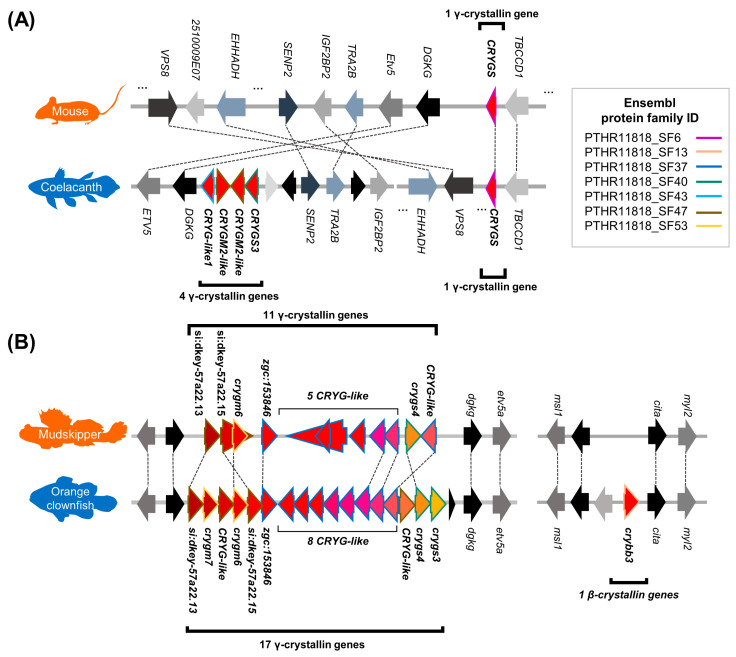
Synteny near the βγ-crystallin coding genes that were contracted in terrestrial vertebrates. Dashed line indicates orthologous relationship. (**A**) Four less γ-crystallin coding genes in mouse genome compared to that of coelacanth with the syntenic support. (**B**) Six less γ-crystallin coding genes (left) and one less β-crystallin coding gene (right) in the mudskipper genome compared to the that of orange clownfish with the syntenic support. Genomic coordinates are available in the Supplementary Table S9.

Next, we investigated the orthology relationship between mouse (representing terrestrial tetrapods) and coelacanth, or between mudskipper and orange clownfish (representing aquatic teleostean fishes) based on Ensembl ortholog and Ensembl protein family. We discovered that there was contraction of βγ-crystallin coding genes including rM and S-crystallin coding genes in the two land-adapting lineages (Figure 5B,C). With these species, we compared the gene order near the βγ-crystallin coding genes between land-adapting and aquatic species with Ensembl ortholog relationship to check whether the contraction of βγ-crystallin coding genes in the land-adapting species was supported by their synteny as well. In the genome of mouse and coelacanth (Figure 6A), *CRYGS* located downstream of *TBCDD1* gene was well-conserved, but the coelacanth genome included additional four γ-crystallin coding genes without mouse orthologs, and the adjacent coelacanth genes were in a different order compared to that of the mouse genome. On the other hand, a comparison between mudskipper and orange clownfish revealed a well-conserved synteny near γ-crystallin genes (Figure 6B, left). Mudskipper was carrying 11 copies of γ-crystallin genes as opposed to 17 copies of orange clownfish. Finally, we compared the synteny of *crybb3* to see if this part of mudskipper genomes is conserved and if this gene is not found in mudskipper. The syntenic relationship near *crybb3* was relatively well-conserved, while *crybb3* gene were not found in mudskipper (Figure 6B, right). Thus, our findings suggest contraction of βγ-crystallin coding genes in the land-adapting lineages.

## 4. Discussion

Mudskippers provide insights into the ongoing transition from aquatic to terrestrial lifestyle, resembling the vertebrate land invasion. We detected the amino acid substitutions in land-adapting lineages with the signature of positive selection in *NOD1*, *ADCY7*, *NOA1*, *LAMB2*, and *KMO* that are involved in immune responses, regulation of mitochondrial oxidative phosphorylation, and development of the kidney. On the other hand, tetrapod-specific and mudskipper-specific PSGs were functionally enriched in DNA repair related processes. Moreover, we discovered that eight gene families were commonly contracting in both tetrapod and mudskipper lineages, including contraction of βγ-crystallin gene superfamily.

The transition from an aquatic to terrestrial environment involves exposure to terrestrial pathogens. In mudskippers, it was proposed that their water-to-land transition is associated with the evolution of innate immune system as a defense mechanism from the terrestrial microbes [23]. Similarly, there have been studies to suggest the importance of immunity in the evolution of tetrapods [61,62], which may be related to the exposure to terrestrial microbes. Cytokines such as tumor necrosis factor act as an important signaling factor to activate the immune responses. Thus, the functional enrichment of “positive regulation of tumor necrosis factor production” among the union PSGs identified by both tetrapod and mudskipper lineages might be associated with the evolution of innate immunity in land-adapting lineages. One of the notable PSG candidates is *NOD1* that encodes a receptor mediating innate and acquired immunity by recognizing lipopolysaccharide (LPS), which is the main component of the outer membrane of Gram-negative bacteria. In human, it was reported that after *NOD1*′s recognition of the pathogenic bacteria’s LPS, *NOD1* activates the NF-κB signal pathway and promotes the expression of inflammatory cytokines [63]. Recently, it was revealed that *NOD1* of teleostean fish also identify LPS and activate the NF-κB signal pathway by recruiting *RIPK2* [51]. Interestingly, Mudskipper possesses the largest number of Toll-like receptor (TLR) 13 coding genes among fish species [23], which belongs to the TLR gene family that synergistically works with *NOD1* to promote production of cytokines involved in innate immunity [64]. Thus, our finding of positive selection on *NOD1* gene might demonstrate the potential role of *NOD1*-mediated immunity along with expansion of TLR genes against pathogenic bacteria in terrestrial environments.

It is also worth paying attention to positive selection in mitochondrial oxidative phosphorylation and kidney development. Oxidative phosphorylation is the pervasively found metabolic pathway to produce ATP by using oxygen as a terminal electron acceptor. Thus, aerial respiration of terrestrial tetrapods and mudskippers might require elaborate regulation of mitochondrial oxidative phosphorylation to adapt to the increased level of oxygen in air compared to water. On the other hand, the kidney in terrestrial vertebrates is responsible for the excretion of nitrogen compounds such as urea or uric acid. However, many fish, including mudskippers, use their gills to excrete waste nitrogen as ammonia. Thus, positive selection in the genes involved in kidney development may not be related to nitrogen waste excretion for mudskippers. The kidney is also important for retaining the water and salt in terrestrial tetrapods. Likewise, in the case of teleostean fish, the kidney acts as a major osmoregulator and this is especially important for mudskippers, which are adapted to the intertidal environment of mudflat with extremely fluctuating salinity. Thus, the signature of positive selection on the genes related to kidney development might be related to osmotic stress in the two independent land-adapting lineages.

In tetrapods and mudskipper, we detected contraction of βγ-crystallin coding gene superfamilies, especially with a smaller number of γ-crystallin coding genes, which were both supported by the CAFE result and Ensembl protein family analysis. The main function of γ-crystallin in vertebrate eye lens is to prevent light scattering with high molecular refractive index increment in the nucleus of lens [65]. Interestingly, it is known that aquatic fish have multiple copies of γ-crystallin genes, while these genes are lost in many terrestrial vertebrates, especially γM-crystallins [66,67,68]. We detected that coelacanth and most aquatic teleostean fish species showed more copies of γM-crystallin genes compared to terrestrial tetrapods and mudskipper. The γM-crystallins, or so-called ‘aquatic’ crystallins [69], were proposed to exist in high density in the lens of aquatic fish to focus on light in water, which has the same refractive index as the cornea [60]. As ancestors of tetrapods adapted to the terrestrial environment, it is thought that the ancestors were required to soften their aquatic lenses and reduce the refractive index to obtain aerial vision that could focus at a distance [70]. Thus, it is believed that there must have been strong selective pressures to remove unnecessary γ-crystallins during vertebrate land invasion [70]. It is worth pointing out that *Periophthalmus* sp. mudskippers, the most land-adapting group among mudskippers, have a flattened lens and a steeply curved cornea, which enable their high-resolution aerial vision [71] unlike the spherical lens of most aquatic fish. Thus, our discovery on the contraction of γ-crystallin coding genes in tetrapods and mudskipper lineages might demonstrate the convergent contraction of unnecessary copy of γ-crystallins during the land adapting process to acquire high resolution aerial vision along with their flattened lens. Nevertheless, whether the loss of γ-crystallin coding genes in the mudskipper or the independent duplications in the aquatic teleost was a more reliable scenario needs more investigation to explain our findings.

In summary, the findings in this study suggest the possibility of similar genetic adaptation of mudskippers to ancient tetrapod for overcoming environmental constraints during the land adaption. Even though it is practically unrealistic to expect identical molecular convergence between mudskippers and terrestrial tetrapods that diverged 435 million years ago, mudskippers have surprisingly similar physiological and behavioral characteristics with terrestrial tetrapods, which suggests that water-to-land transition would have acted as an external force to drive the genetic change of genes associated with similar biological processes. Consistent with our findings, several studies have also proposed essential adaptations of ancestral tetrapods associated with their terrestriality including evolution of the kidney [48], changes in the immune system [61,62], and acquirement of aerial visions [70]. In this regard, the findings in our study could be the evidence of such essential changes in the land adaptation of the two lineages. Even though we conducted bioinformatics analyses to detect the evolutionary signals of amino acid substitutions and gene families based on the reference assembly and its annotation, experimental validation including RNA-seq, reference-quality genome sequences of other mudskippers or closely related species, search of gene loss (Supplementary Table S10), and validation of functional changes of protein products is necessary to check the biological interpretation of these findings and for further research. Thus, more studies and validation are needed to determine whether mudskipper is a proper model system to study genomic signature of land adaptation of ancient tetrapods.

## Figures and Tables

**Table 1:** Species information used in this study.

Scientific Name	Common Name	Taxon ID	Classification	Land Adaptation	Ensembl Assembly
*Periophthalmus magnuspinnatus*	Giant-fin mudskipper	409849	infraclass Teleostei	O	PM.fa
*Homo sapiens*	Human	9606	superclass Tetrapoda	O	GRCh38.p13
*Mus musculus*	Mouse	10090	superclass Tetrapoda	O	GRCm38.p6
*Bos taurus*	Cattle	9913	superclass Tetrapoda	O	ARS-UCD1.2
*Gallus gallus*	Chicken	9031	superclass Tetrapoda	O	GRCg6a
*Latimeria chalumnae*	Coelacanth	7897	Non-tetrapod Sarcopterygii	X	LatCha1
*Danio rerio*	Zebrafish	7955	infraclass Teleostei	X	GRCz11
*Astatotilapia calliptera*	Eastern happy	8154	infraclass Teleostei	X	fAstCal1.2
*Amphiprion percula*	Orange clownfish	161767	infraclass Teleostei	X	Nemo_v1
*Oryzias latipes*	Japanese medaka	8090	infraclass Teleostei	X	ASM223471v1
*Parambassis ranga*	Indian glassy fish	210632	infraclass Teleostei	X	fParRan2.1
*Poecilia formosa*	Amazon molly	48698	infraclass Teleostei	X	Poecilia_formosa-5.1.2

## Data Availability

The data presented in this study are openly available in Ensembl 2019 at https://doi.org/10.1093/nar/gky1113, reference number 32.

## Supplementary Materials

The following are available online at https://www.mdpi.com/2076-2615/11/2/584/s1, Table S1: List of tetrapod model PSGs, Table S2: List of mudskipper model PSGs, Table S3: List of tetrapod-and-mudskipper model PSGs, Table S4: Exclusive amino acid substitutions under positive selection (EAASPS, TAAS + Branch-site model of CodeML), Table S5: ClueGO results of mudskipper-specific PSGs, Table S6: ClueGO results of tetrapod-specific PSGs, Table S7: Rapidly contracted gene families in both tetrapod and mudskipper lineages, Table S8: Number of genes in the Ensembl protein families defined as a single gene family by CAFE, Table S9: Genomic coordinates of crystallin gene synteny analysis, Table S10: List of aquatic fish specific orthologues absent in tetrapods and mudskipper. Figure S1: Concept of target-specific amino acids substitution (TAAS) analysis. Figure S2: Detection of EAASPS (mudskipper model), Figure S3: Detection of EAASPS (tetrapod model), Figure S4: Detection of EAASPS (tetrapod-and-mudskipper model), Figure S5: Protein-protein network of PSGs involving in both “DNA repair” and “response to UV”.

## Abbreviations

The following abbreviations are used in this manuscript:

PSG	Positively selected gene
PSS	Positively selected site
BEB	Bayes empirical Bayes
TAAS	Target-specific amino acid substitution
EAASPS	Exclusive amino acid substitution under positive selection
FDR	False discovery rate
