# Supplementary figures and images for: Genetic Adaptations in Mudskipper and Tetrapod Give Insights into Their Convergent Water-to-Land Transition
Source: Animals (Basel). 2021 Feb 23;11(2):584. doi: 10.3390/ani11020584 (PMC7926366; doi:10.3390/ani11020584)

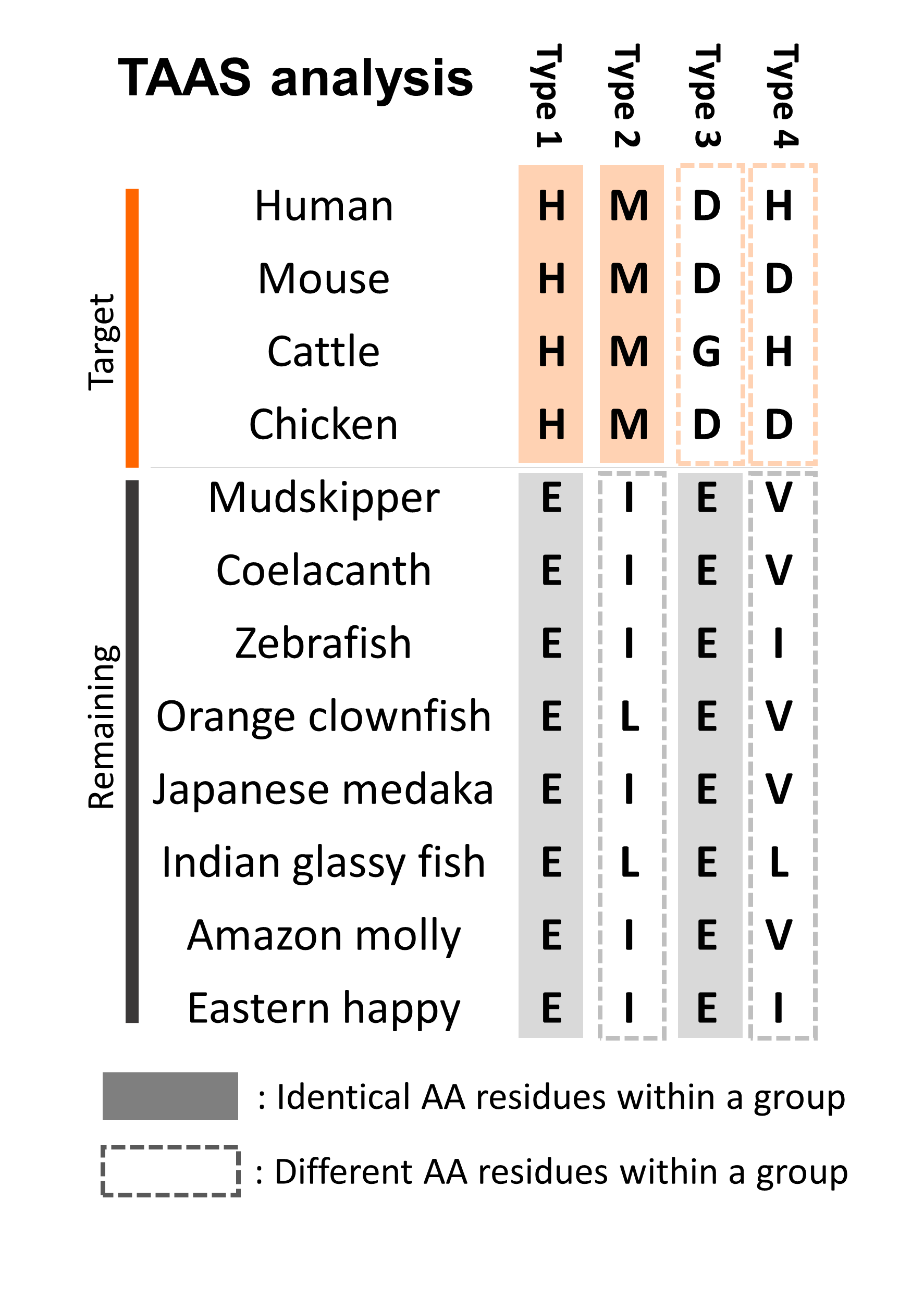

Supplement: Supplementary file 1 [file animals-11-00584-s001.zip › Figure S1.tif]

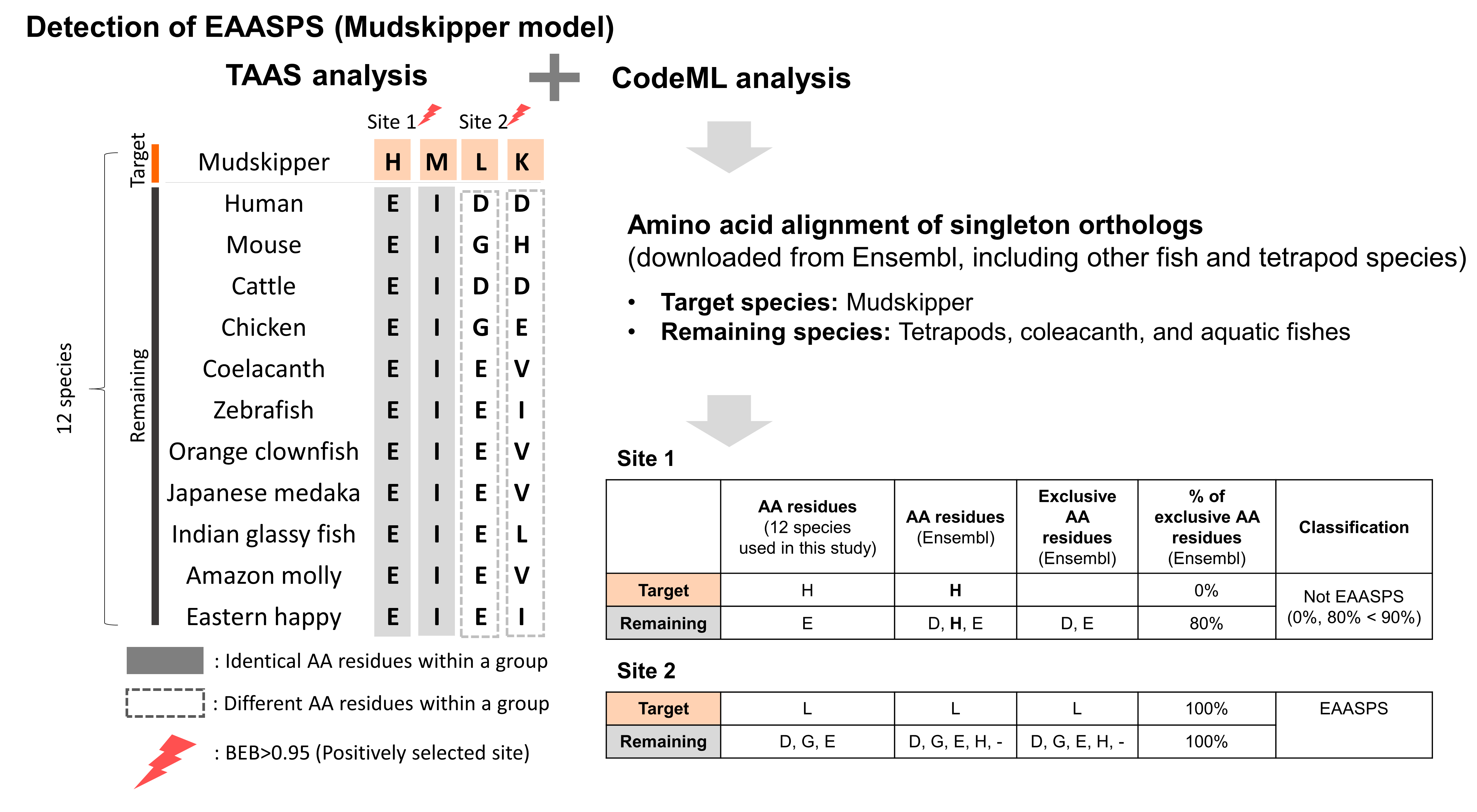

Supplement: Supplementary file 1 [file animals-11-00584-s001.zip › Figure S2.tif]

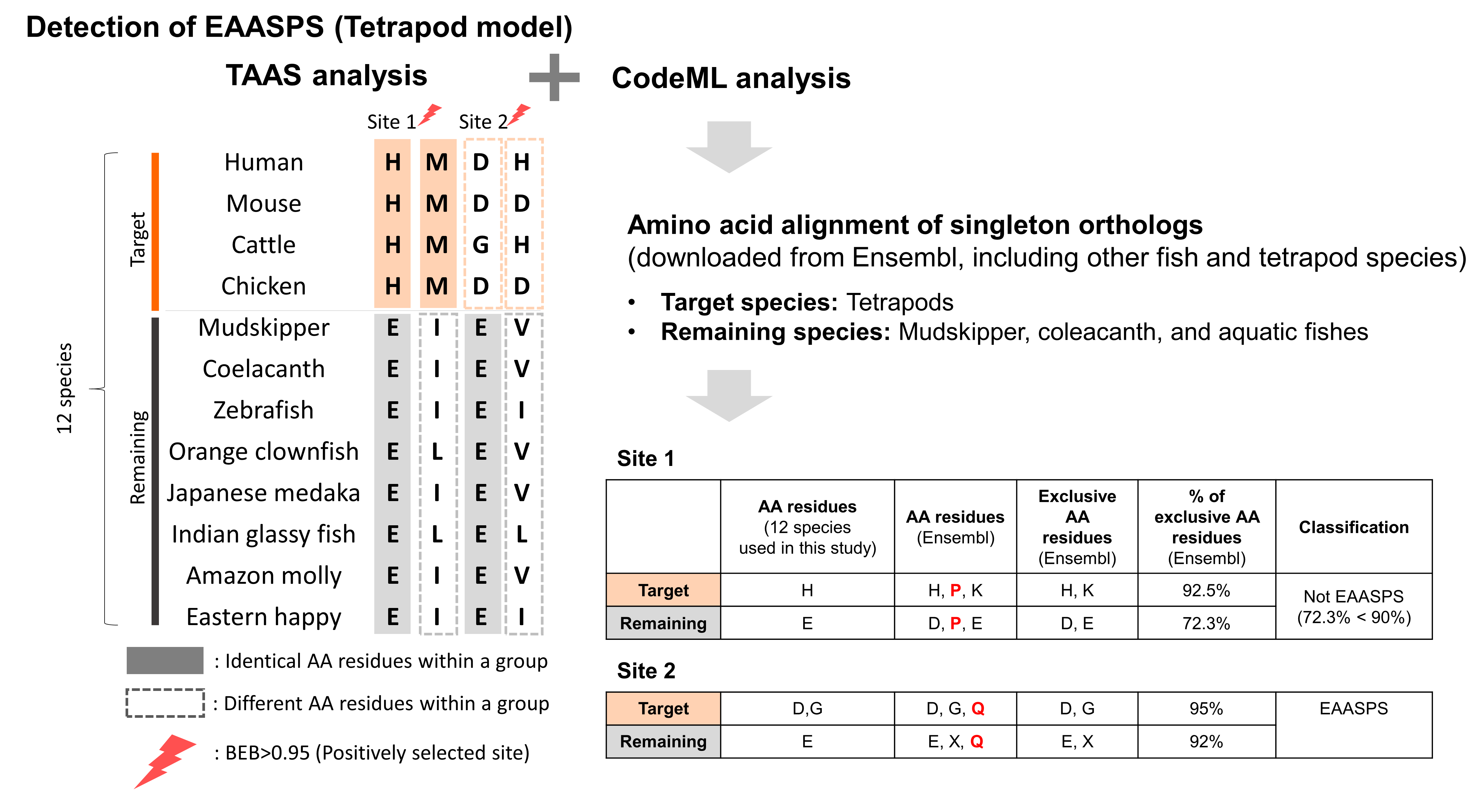

Supplement: Supplementary file 1 [file animals-11-00584-s001.zip › Figure S3.tif]

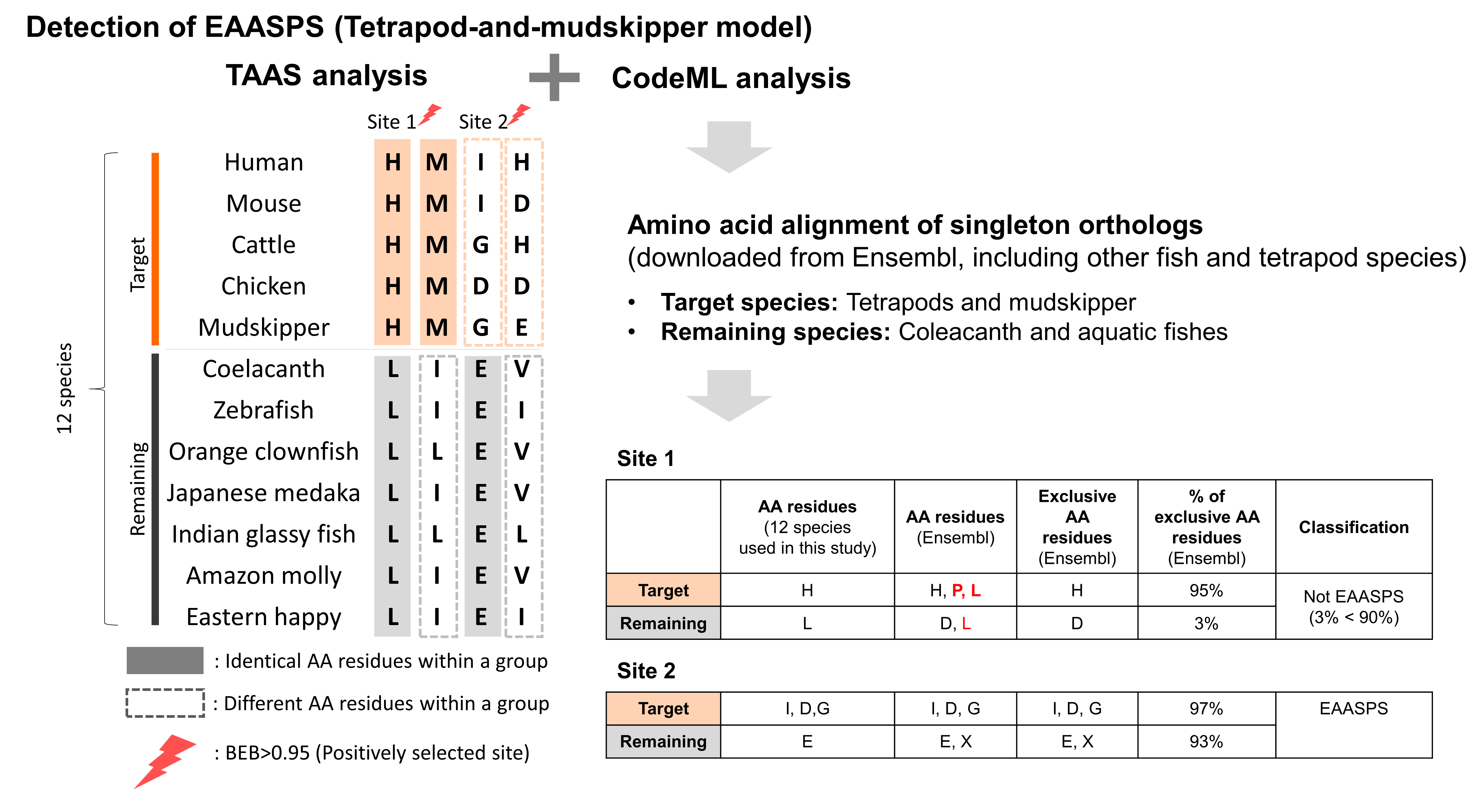

Supplement: Supplementary file 1 [file animals-11-00584-s001.zip › Figure S4.tif]

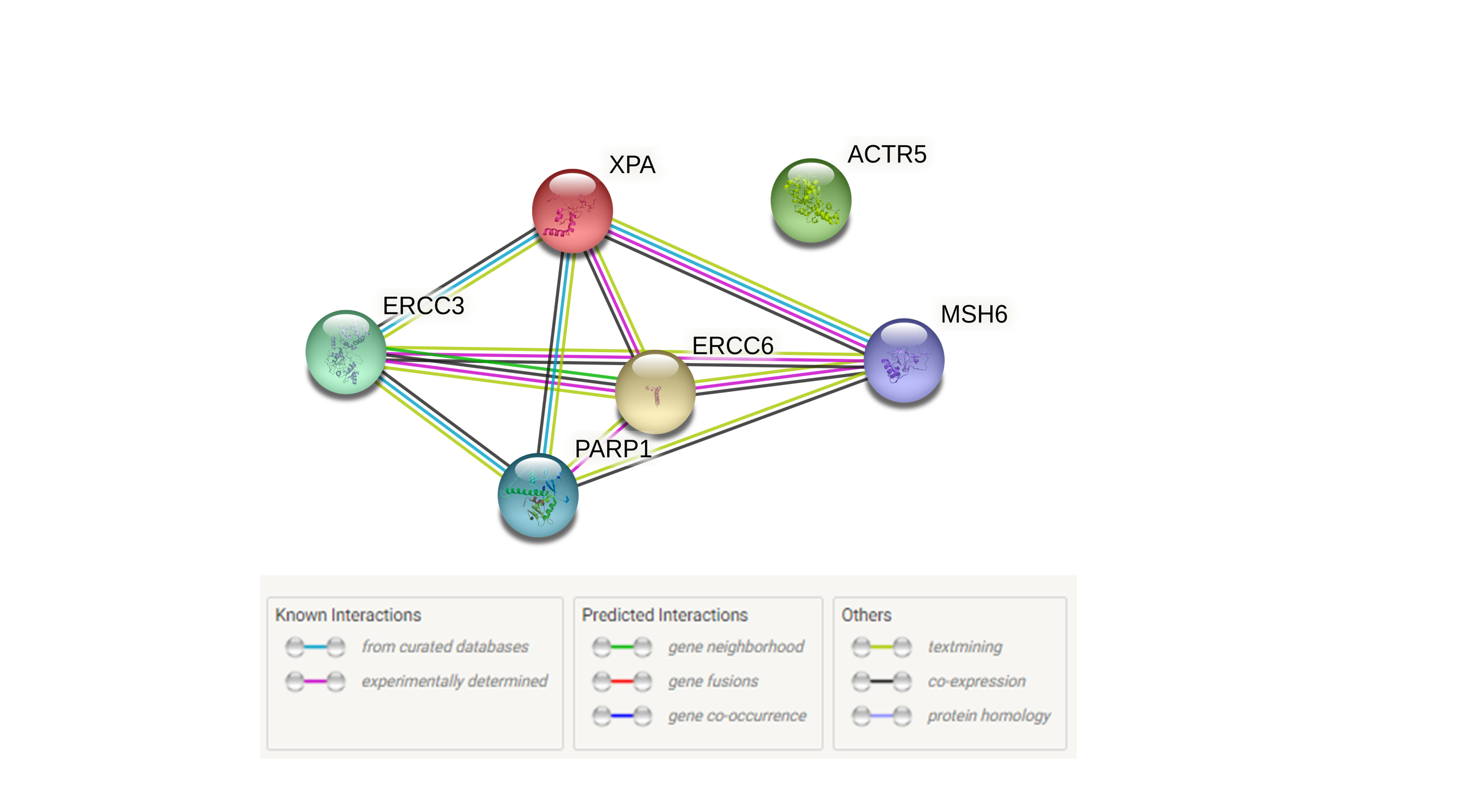

Supplement: Supplementary file 1 [file animals-11-00584-s001.zip › Figure S5.tif]
